# LzSCL9, a Novel GRAS Transcription Factor in Lanzhou Lily (*Lilium davidii* var. *unicolor*), Participates in Regulation of Trichokonins-Primed Heat Stress Tolerance

**DOI:** 10.3390/plants13162330

**Published:** 2024-08-21

**Authors:** Xing Cao, Liping Ding, Jiahui Liang, Yanrong Zhou, Xiulan Chen, Haiyan Li, Tao Liu, Wenxiu Yue, Juanjuan Sui, Liangbao Jiang, Yulian Qian, Dongdong Yang, Bo Wang, Hailing Zhang, Ze Wu, Xiaoyan Song

**Affiliations:** 1 College of Architecture, Yantai University, Yantai 264005, China; caoxinglina@163.com (X.C.);; 2 Key Laboratory of Landscaping Agriculture, Ministry of Agriculture and Rural Affairs, College of Horticulture, Nanjing Agricultural University, Nanjing 210095, China; 3 Jiangsu Key Laboratory for Horticultural Crop Genetic Improvement, Institute of Pomology, Jiangsu Academy of Agricultural Sciences, Nanjing 210014, China; 4 State Key Laboratory of Microbial Technology, Marine Biotechnology Research Center, Shandong University, Qingdao 266237, China; 5 College of Life Sciences and Medicine, Zhejiang Sci-Tech University, Hangzhou 310018, China; 6 Engineering Technology Research Center of Anti-Aging Chinese Herbal Medicine, Biology and Food Engineering College, Fuyang Normal University, Fuyang 236037, China; 7 College of Life Science, Yantai University, Yantai 264005, China

**Keywords:** Lanzhou lily, trichokonins, thermotolerance, GRAS, LzSCL9

## Abstract

In our previous research, we found that trichokonins’ (TKs) employment improved the thermotolerance of the Lanzhou lily, a renowned edible crop species endemic to China that is relatively susceptible to high temperatures (HTs). Here, a novel Lanzhou lily GRAS gene, *LzSCL9*, was identified to respond to heat stress (HS) and HS+TKs treatment based on transcriptome and RT-qPCR analysis. TKs could improve the upregulation of *LzSCL9* during long-term HS. The expression profile of *LzSCL9* in response to HS with or without TKs treatment showed a significant positive correlation with *LzHsfA2a-1*, which was previously identified as a key regulator in TKs’ conferred resilience to HT. More importantly, overexpression of *LzSCL9* in the lily enhanced its tolerance to HTs and silencing *LzSCL9* in the lily reduced heat resistance. Taken together, this study identified the positive role of LzSCL9 in TK-induced thermotolerance, thereby preliminarily establishing a molecular mechanism on TKs regulating the thermostability of the Lanzhou lily and providing a new candidate regulator for plant heat-resistant breeding.

## 1. Introduction

Recently, global warming, especially extremely high temperatures (HTs), has led to crop yield losses and a food security crisis [1]. Owing to their sessile nature, plants have evolved complex mechanisms to sense and react to heat stress (HS). The sophisticated signaling networks underlying the heat stress response (HSR) involve phytohormones, second messengers, regulatory proteins, functional proteins, and non-coding RNAs. Arabidopsis EARLY FLOWERING 3 (ELF3) and THERMO-WITH ABA-RESPONSE 1 (TWA1) are identified as plant thermosensors [2,3]. Several transcription factors (TFs), e.g., heat shock transcription factor (HSF), DREB, WRKY, MYB, NAC, BBX, bZIP, HD-Zip, bHLH, and GRAS have been reported to play vital roles in obtaining thermotolerance [4,5,6,7,8,9,10,11,12,13,14]. The plant-specific GRAS family is named after its initial three members: GAI (gibberellic acid-insensitive), RGA (repressor of GAI), and SCR (scarecrow). Most GRASs possess a highly conserved C-terminal GRAS domain containing leucine heptad repeat I (LHR I), VHIID, leucine heptad repeat II (LHR II), PFYRE and SAW motifs, and a variable N-terminal domain [15]. The GRAS domain is indispensable for either protein–DNA or protein–protein interactions, while the N-terminal part of GRAS seems to be necessary for molecular recognition [16,17,18,19,20].

To date, a total of 34, 60, 153, 106, 150, 54, 117, 49, and 59 GRAS genes are found in *Arabidopsis thaliana*, *Oryza sativa*, *Triticum avestum*, *Populus simonii*, *Gossypium hirsutum*, *Solanum lycopersicum*, *Glycine max*, *Liriodendron chinense*, and *Rosa chinensis*, respectively, based on genome-wide analyses [21,22]. The GRASs in Arabidopsis are classified into ten subfamilies: DELLA, DLT, SCR, LAS, SHR, HAM, PAT1, SCL (SCR-like) 3, SCL4/7, and LlSCL (SCL9) [20]. There is growing evidence that GRAS proteins play diverse and crucial roles in regulating plant growth and development [19,23]. AtSCL15 interacts with HDA19 to repress the seed maturation programme in Arabidopsis [24]. HAM is essential for shoot meristem maintenance of Petunia [25]. The SHR-SCR module initiates cortical cell division for nodule organogenesis and accommodation of rhizobia in legumes [26]. DELLA proteins AtRGL1, AtRGL2, and AtRGA work together to suppress stamen and anther development in plants lacking GA [27]. GRASs are covered to take part in plant responses to abiotic stresses, e.g., drought stress, low and high temperatures, and salt stress. HcSCL13 isolated from *Halostachys caspica* enhances salt stress tolerance in transgenic Arabidopsis [28]. The apple miR171i-SCL26.1 module improves resistance to drought stress via regulating ascorbic acid metabolism and antioxidant gene expression [29]. VaPAT1 is involved in cold stress response in grapes by regulating JA biosynthesis [30]. PgGRAS52 and PgGRAS55 are strongly upregulated under heat stress in Pearl millet [31]. Cabbage BoSCL13 are more strongly expressed in heat-resistant groups than in heat-sensitive groups, suggesting SCL13 as an early selection marker for heat-resistance breeding [32]. JrGRAS2 of *Juglans regia* results in an elevated heat tolerance of transgenic yeasts [33].

The Lanzhou lily (*Lilium davidii* var. *unicolor*) is a Chinese specialty with highly comestible, medicinal, and ornamental value. It mainly grows in a mountain area at an elevation of 1800–2600 m, which is located in the south of Lanzhou City, Gansu province, China [34]. This area has an arid and cool climate, facilitating the growth of Lanzhou lilies. However, due to the greenhouse effect, HTs in summer have become a major limitation to the yield and landscape quality of the Lanzhou lily in recent years. Breeding (traditional breeding or genetic engineering), protectant (plant growth regulator, osmolytes, microbes, mineral nutrients, or soil amendments) application, or proper agricultural practices are useful to overcome this constraint. Lily cultivar ‘Sunflower’ bred from the hybrid offspring of Asiatic lily ‘Valdisole’ and Oriental lily ‘Siberia’ shows strong heat resistance [35]. TFs HSF, WRKY, DREB, NAC, MYB, ERF, and HD-Zip are proved to be positive regulators in lily thermotolerance establishment [12,36,37,38,39,40,41], but GRAS has not been reported to be HS signaling. Pretreatment of CaCl_2_ or salicylic acid (SA) enhances the thermotolerance of ornamental lilies [42,43], and we reported that the application of trichokonins (TKs) secreted from *Trichoderma longibrachiatum* SMF2, which is known as a biocontrol of fungi, increased the heat tolerance of the Lanzhou lily [44]. The preliminary study revealed that TKs promoted the abilities of water retention and photosynthesis, the activity of antioxidant enzymes, and the level of HS-associated phytohormones and HS-protective genes under HS [44]. *LzHsfA2a-1* was identified as a potential key gene associated with Lanzhou lily thermotolerance conferred by TKs. In this study, to further elucidate the mechanism of TKs on regulating the HS tolerance of the Lanzhou lily, we isolated and identified a heat- and heat + TK-inducible GRAS gene, *LzSCL9*, from the Lanzhou lily based on the differently expressed genes (DEGs) in the HS transcriptome data with or without TKs treatment. Subsequently, the function of LzSCL9 in the TK-mediated Lanzhou lily response to HS was analyzed.

## 2. Results

### 2.1. GRAS Gene Expression Was Significantly Regulated by TK Treatment under HS

**Figure 1:**
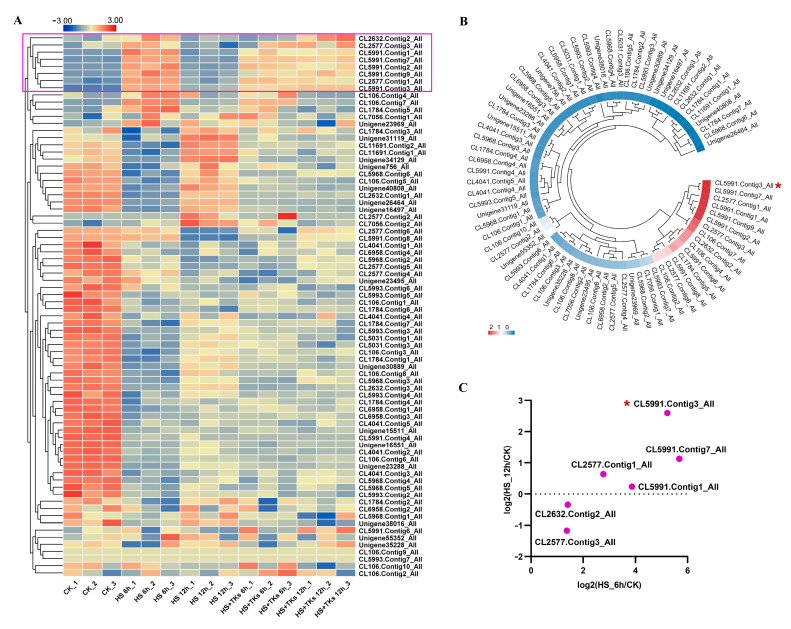
The analysis of *GRASs* expression in reaction to HS or HS + TKs treatment based on transcriptome data. (**A**) *GRASs* expression in response to HS or HS + TKs treatment. Eight genes (highlighted with pink rectangular box) were upregulated under 12 h of HS in the presence of TKs. (**B**) Heat map of correlation between *LzHsfA2a-1* and 75 *GRAS* gene expression levels. Pearson’s correlation (r) analyses (*p* < 0.05) were used. CL5991.Contig3_All showed the highest correlation with *LzHsfA2a-1* in gene expression. (**C**) Expression analysis of 6 differentially expressed *GRAS* genes at different time points under HS treatment using RNAseq. CL5991.Contig3_All (highlighted with red asterisk) was selected for further study.

TFs are DNA-binding proteins that regulate downstream gene expression and have been increasingly recognized to be important contributors to the improvement of plant thermotolerance [4,5,6,7,8,9,10,11,12,13,14]. Our previous research showed that many TFs, such as HSF, bHLH, NAC, MYB, C3H, WRKY, AP2-EREBP, and GRAS were differentially expressed in answer to TK treatment under HS [44]. Here, we choose GRAS, which has not been documented to function in lily HSR, for further study. Among the 55 differentially expressed *GRAS* genes, 10 genes were upregulated in response to 6 h of HS, and then most of the expressions showed a marked drop under long-term (12 h) HS (Figure 1A). However, in the presence of TKs, eight of these ten genes were upregulated under long-term HS, including a *DELLA* gene (CL2632.Contig2_All) and *LlSCL* genes (CL2577.Contig1_All, CL2577.Contig3_All, CL5991.Contig1_All, CL5991.Contig2_All, CL5991.Contig3_All, CL5991.Contig7_All, CL5991.Contig9_All) (Figure 1A). Considering the key role of LzHsfA2a-1 in the acquisition of TK-induced thermotolerance of the Lanzhou lily [44], we also analyzed the gene expression correlation between *LzHsfA2a-1* and 75 *GRAS* genes. The results revealed that all eight genes showed a significant correlation with *LzHsfA2a-1*, with CL5991.Contig3_All having the highest correlation *p*-value (Figure 1B). CL5991.Contig2_All and CL5991.Contig9_All were not considered due to their extremely low FPKM values (<0.01) in the control samples (CK). Thus, among the other six genes, CL5991.Contig3_All (log2(HS_12h/CK) = 2.60, log2(HS_6h/CK) = 5.2) was selected for further investigation of the function in TK-induced thermotolerance (Figure 1C).

### 2.2. Cloning and Sequence Analysis of LzSCL9

**Figure 2:**
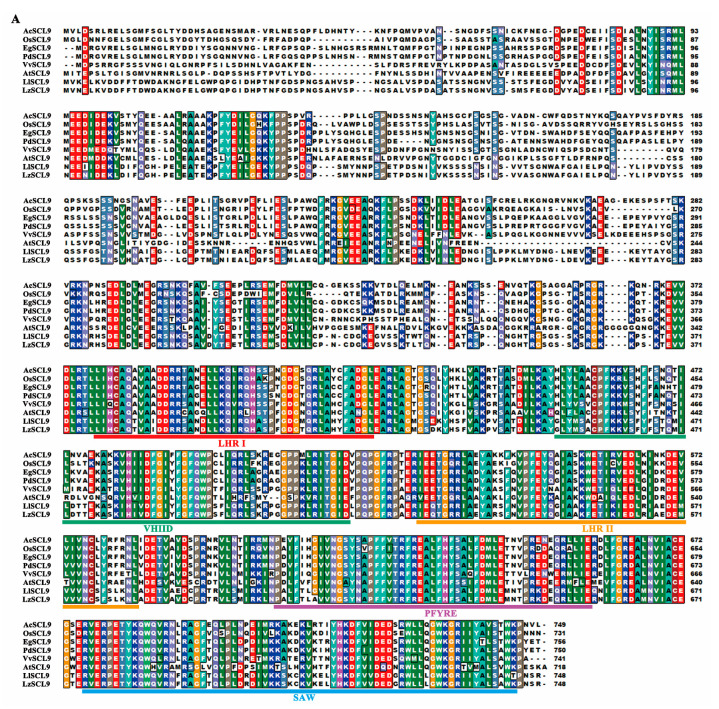

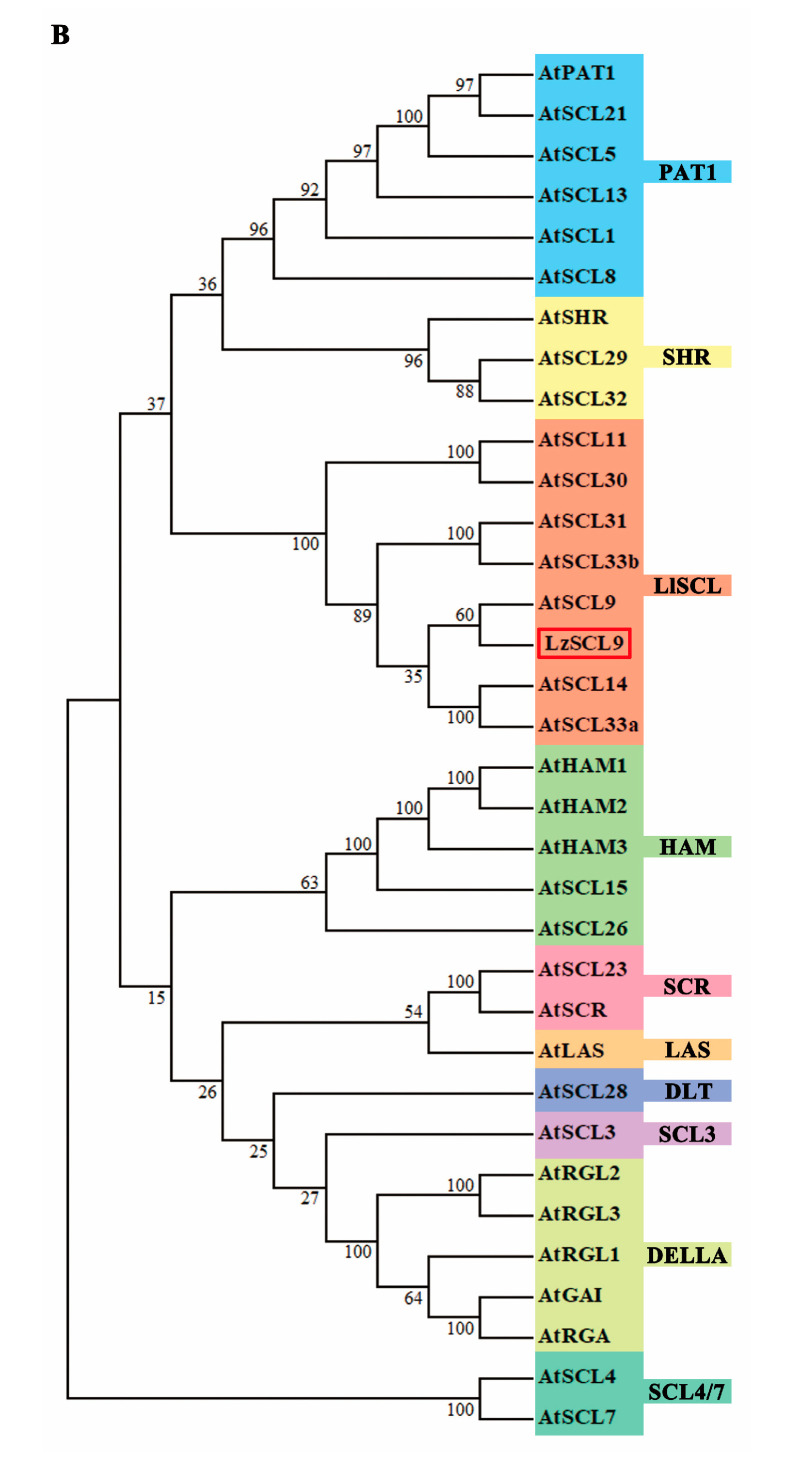
Phylogenetic relationship and sequences alignment of SCL9 in various species. (**A**) Multiple alignments of LzSCL9 with other plant SCL9. LHR I, VHIID, LHR II, PFYRE, and SAW motifs are indicated by horizontal lines. (**B**) Phylogenetic tree analysis of LzSCL9, LlSCL9, and GRASs from Arabidopsis was fulfilled using the neighbor-joining method with 1000 bootstrap replicates. LzSCL9 was highlighted with a rectangular box. Ac: *Ananas comosus*, Os: *Oryza sativa*, Eg: *Elaeis guineensis*, Pd: *Phoenix dactylifera*, Vv: *Vitis vinifera*, At: *Arabidopsis thaliana*, Ll: *Lilium longiflorum*.

According to the transcriptome analysis, we chose the speculative *LlSCL* (CL5991.Contig3_All) gene for further study. The open reading frame (ORF) of *LzLlSCL* was 2247 bp, which encoded a deduced protein of 748 amino acids (Figure 2A). Phylogenetic analysis between all 34 Arabidopsis GRASs and LzLlSCL demonstrated that LzLlSCL was classified into the LlSCL (SCL9) subfamily and was closely related to AtSCL9 (Figure 2B), so it was formally named *LzSCL9* (PP782570). Multiple alignments were performed among LzSCL9 and SCL9 in *Lilium longiflorum*, *Arabidopsis thaliana*, *Ananas comosus*, *Elaeis guineensis*, *Oryza sativa*, *Phoenix dactylifera*, and *Vitis vinifera*. LzSCL9 (XAT94018) showed a 41.94% similarity to AtSCL9 and a 97.99% similarity to LlSCL (BAC77269.2), which was implicated in the transcriptional regulation of microsporogenesis in lily anther [45]. Sequence analysis indicated that the typical GRAS functional domains containing LHR I, VHIID, LHR II, PFYRE, and SAW motifs were found in the deduced amino acid sequence of LzSCL9.

### 2.3. LzSCL9 Transcript Was Induced by HS and HS + TKs Treatment

**Figure 3:**
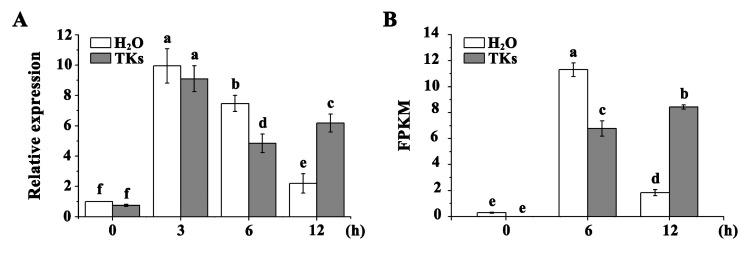
qRT-PCR analysis of *LzSCL9* expression and validation of the DEG. (**A**) Relative expression of *LzSCL9* in leaves under 2 mg/L TKs treatment or distilled water at 40 °C HS for different durations (0, 3, 6, and 12 h). (**B**) FPKM of *LzSCL9* (CL5991.Contig3_All) from RNA-seq. Different lowercase letters represent significant differences at a 0.05 level with Duncan’s multiple range test.

The expression levels of *LzSCL9* responding to HS and TKs treatment were detected using RT-qPCR. Compared to RT (22 °C), *LzSCL9* expression was induced by 40 °C HS, peaking after 3 h, followed by a gradual decline (Figure 3A). Compared to HS, HS + TKs treatment had no significant effect on *LzSCL9* expression in short-term HS (3 h) but caused induction in long-term HS (12 h) (Figure 3A). The expression profile of *LzSCL9* coincided with data obtained from RNA-Seq (Figure 3A,B). The findings suggested that TK application could improve the upregulation of *LzSCL9* under long-term HS.

### 2.4. LzSCL9 Overexpression Enhanced the Thermotolerance of Lily

**Figure 4:**
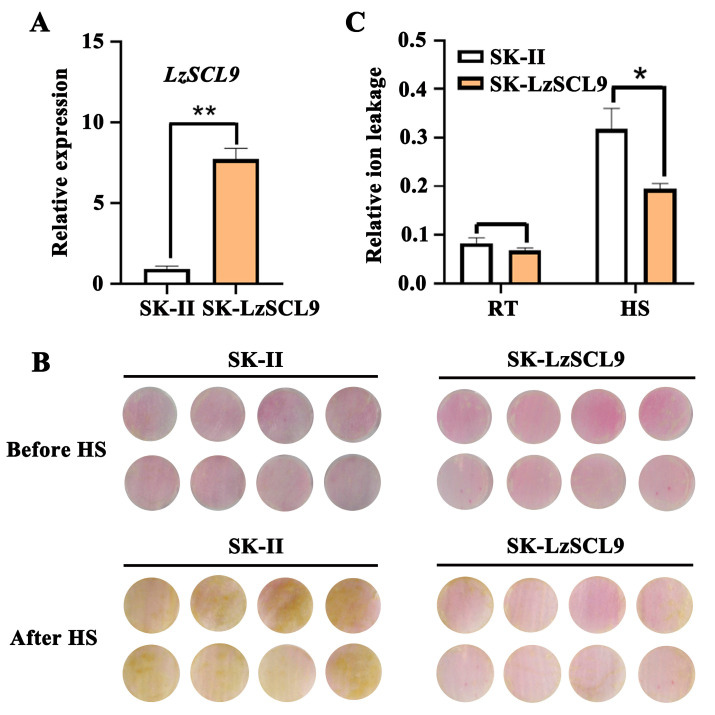
Thermotolerance assessment in lily petal discs overexpressing *LzSCL9*. (**A**) Detection of *LzSCL9* expression in *LzSCL9* overexpression petal discs. (**B**) Petal disc color phenotypes observed at 22 °C (RT) and after 12 h exposure to 40 °C (HS). (**C**) Relative ion leakage of petal discs at RT (22 °C) and following 12 h HS (40 °C). Data were analyzed using Student’s *t*-test (* *p* < 0.05, ** *p* < 0.01).

To study the in vivo role of LzSCL9, *LzSCL9* was overexpressed in lily petals through transient transformation (Figure 4). The RT-qPCR analysis indicated that *LzSCL9* was overexpressed at a higher level compared to SKII-control (Figure 4A). The injury mechanisms under HTs involve the petals fading in phenotype and increased electrolyte leakage in physiology. After HS treatment, petals with transient overexpression of *LzSCL9* displayed less fading than the control group (Figure 4B). Additionally, the overexpression of *LzSCL9* in petal discs did not impact the relative ion leakage at RT (22 °C). Nevertheless, the value in the *LzSCL9* overexpression discs was significantly lower than that in the control discs following HS (Figure 4C). These results showed that *LzSCL9* overexpression alleviated the lily cell damage caused by HS and increased their thermotolerance.

### 2.5. LzSCL9 Silencing Reduced the Thermotolerance of Lily

**Figure 5:**
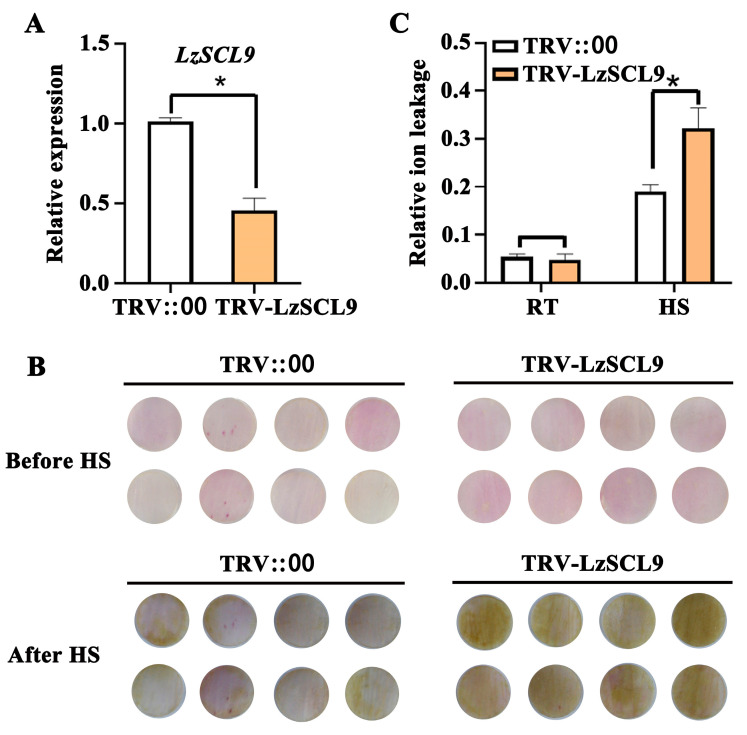
Evaluation of thermotolerance in *LzSCL9*-silenced lily petal discs. (**A**) *LzSCL9* expression level in TRV-VIGS petals. (**B**) The color phenotypes of petal discs at 22 °C (RT) and subjected to 40 °C for 12 h (HS). (**C**) Relative ion leakage of petal discs at RT and following HS. Data were processed using Student’s *t*-test (* *p* < 0.05).

To further explore the role of LzSCL9 in the heat tolerance of lily, *LzSCL9* expression was silenced in petal discs using TRV-VIGS (Figure 5). The silencing of *LzSCL9* led to lower gene expression in contrast to the TRV2 control via RT-qPCR analysis (Figure 5A). It was seen that petals deficient in *LzSCL9* exhibited more fading after heat stress in comparison with the TRV2 controls (Figure 5B). In addition, the silencing of *LzSCL9* in petal discs had no influence on the relative ion leakage at 22 °C; yet, post-HS, the value of TRV2-LzSCL9 discs was notably higher than that of the TRV2 control discs (Figure 5C). These results indicate that the silencing of *LzSCL9* worsened the damage generated by HS in lily cells and decreased thermotolerance.

## 3. Discussion

Unlike breeding, including conventional breeding and transgenic breeding, the employment of protectants in the form of plant growth regulators, osmolytes, microbes, mineral nutrients, polypeptides, or soil amendments have been discovered to be quickly effective in alleviating HS-caused lesions in plants. SA is beneficial to improving heat resilience in Arabidopsis, rice, peas, lilies, and grapevines [43,46,47,48,49], while epibrassinolide induces heat resistance in melon, tomato, and tea trees [50,51,52]. Treatment with melatonin confers thermotolerance in tall fescue, perennial ryegrass, wheat, and Chinese cabbage [53,54,55,56]. Some studies have suggested that beneficial microorganisms associated with plants synergistically improve plant growth and stress tolerance, and this application is eco-friendly, easily available, and sustainable [57,58]. Plant growth-promoting rhizobacteria or fungi enhance plant tolerance to abiotic stresses by producing phytohormones and other secondary metabolites and elevating nutritional status. Only a few research studies have addressed plant growth-promoting microbes (PGPMs) or elicitors secreted from PGPMs tackling HS in plants. Arbuscular mycorrhizal inoculation enhances the tolerance to HS in tomato, pepper, and cucumber [59]. The defense response of Arabidopsis induced by *Pst* DC3000 mitigates the damage caused by HS [60]. The compound 2-Amino-3-methylhexanoic acid sourced from *Alternaria alternata* contributes to HT tolerance by mitigating physiological injury in tea plants [61]. The Lanzhou lily is sensitive to HTs, so the increasing frequency of HTs in summer has become the primary abiotic factor limiting its production and introduction. Since the breeding of Lanzhou lilies against HS progressed very slowly, the application of PGPMs or inducers generated by PGPMs seems a promising approach in the enhancement of heat tolerance. TKs are peptaibols isolated from *Trichoderma longibrachiatum* SMF2 and composed of isoform A (20-aa) and isoform B (11-aa) [62]. Our previous studies mainly focused on its broad-spectrum antimicrobial activity and the induction of systemic resistance in plants [62,63]. Firstly, we recently reported the role of TKs on plant abiotic stresses. We found that TKs increased the tolerance of the Lanzhou lily to HTs, as evidenced by lower mortality after HS [44]. Subsequent findings revealed that HsfA2a probably plays a core role in heat resistance primed by TKs, and similar discoveries were observed in Arabidopsis, wheat, and grapevines that HsfA2 is a key effector downstream of heat sensing [64,65,66,67]. A number of TFs are identified as upstream regulators of HSFA or interactors with HSFA in plant HSR. The transcriptional upregulation of HsfA2 and HSPs depended on TWA1, which is a temperature sensor in Arabidopsis [3]. DREB2C and ERF95/ERF97, respectively, function as transcriptional activators of HsfA3 and HsfA2 during HSR in Arabidopsis [68,69]. HsfA6B is activated by bZIP60, which links the unfolded protein response to the HSR in maize [11]. The transcriptional cascade modules LlWRKY22-LlDREB2B-LlHsfA3, LlHB16-LlHsfA2-LlMBF1c, LlNAC014-LlDREB2-LlHsfA3, LlWRKY39/LlCaM3-LlMBF1c, LlMYB305-LlHSC70, and LlERF012/LlHsfA1-LlHsfA2 in *Lilium longiflorum* positively participate in the establishment of thermotolerance [12,37,38,39,40,41,70]. BES1 interacts with HsfA1 to increase the HS tolerance of Arabidopsis [71], while lily LlHsfA1 interacts with LlHsfA2 to improve the thermostability of transgenic Arabidopsis [36]. GRASs are also responsive to HS [31,32,33,72] and regulatory mechanisms may be involved in the HSF-HSP pathway [14,73]. JrGRAS2 contributes to the heat tolerance of walnut by regulating Dof transcription and promoting *HSP* expression [73]. Tomato SlGRAS4 enhances high temperature stress tolerance through HSF and ROS signal transduction [14]. Several TFs, such as bHLH, MYB, WRKY, NAC, HSF, HD-Zip, Znf, AP2/ERF, and GRAS are responders under HS or HS + TKs treatment on account of a RNA-Seq analysis [44], and most of these TFs are regulators in lily thermotolerance on the basis of our preliminary research [12,37,38,39,40,41]. Nevertheless, GRAS has not been reported to function in lily HSR so far. Additionally, the expression of several *GRAS* genes showed a significant correlation with *LzHsfA2a-1*. Therefore, GRAS, which might be involved in the HSF-HSP pathway in response to HS, was selected as the research object in the present study.

Evolutionary analyses of GRAS in angiosperms demonstrated that GRAS can be divided into 17 subfamilies: SCR, SHR, NSP1, NSP2, LS, HAM, DELLA, PAT, RAD1, RAM1, DLT, SCLA, SCLB, SCL3, SCL4/7, SCL32, and LlSCL [15]. AtSCL9, AtSCL11, AtSCL14, AtSCL30, AtSCL31, AtSCL33a, and AtSCL33b belong to LlSCL, named after the gene in *Lilium longiflorum* [45,74]. In the current work, LzSCL9 isolated from Lanzhou lilies contained a typical GRAS domain and showed a high similarity to the LlSCL that is responsible for the transcriptional regulation of microsporogenesis in lilies [45]. GRAS members are determined in more and more plants on the basis of genome-wide analyses, in which some *GRASs* are high responders in HSR. For example, *LlSCL* expression in *Liriodendron chinense* is upregulated within 1 h of HS [75]. HS specifically upregulates *RsSCL23* and *RsSHRa* expression in radish [76]. A total of 47 GRAS *genes* are identified from *Dendrobium catenatum*, of which three genes are induced by HS in stems [77]. Our results obtained from the transcriptome analysis suggested that several HT-induced *LzGRAS* genes were further promoted by TKs, and the RT-qPCR analysis confirmed that *LzSCL9* might be a crucial signal transduction component in TK-mediated HSR. The function analysis revealed that GRAS play diverse roles in light and GA signaling, root and shoot formation, fruit ripening, and biotic and abiotic stress responses [19]. However, fewer literatures have verified the function of GRAS in plant thermotolerance. The overexpression of *JrGRAS2* in Arabidopsis and walnut enhances HS tolerance [73]. Silencing *SlGRAS4* in tomato reduced thermotolerance [14], which coincides with the outcome from this work that silencing *LzSCL9* in lily decreased resistance to HS. On the other hand, the thermotolerance of lily was promoted by *LzSCL9* overexpression characterized by a reduced sensitivity to HS. The findings above uncover that LzSCL9 participates in Lanzhou lily adaptation to high temperature stress. One of the consequences of HS is the generation of oxidative stress leading to lipid peroxidation and increased electrolyte leakage. HT stress also influences the content and distribution of plant pigments, mainly consisting of chlorophyll, anthocyanin, xanthophyll, and carotenoid, which results in the color fading from leaves and petals. So, consistent with other studies, the relative ion leakage and petal color fading was chosen as damage indicators for physiology and phenotype, respectively, in this study.

This is the first report where GRAS takes part in the thermotolerance acquisition in lilies. Nevertheless, the mechanism of LzSCL9 on modulating thermotolerance needs to be further explored. GRAS has been described to be regulated by both genetic and epigenetic mechanisms [19,78,79] which provide reference for a follow-up exploration on LzSCL9. The HSR molecular networks with class A HSF as the core have been presented in plants [80], so the involvement of LzSCL9 in HSFA-regulated HS responses will be the focus in our following study. Actually, there is evidence that LzSCL9 participates in the regulation of TK-induced thermostability of the Lanzhou lily via the HSF-HSP pathway, but this needs to be further verified.

## 4. Materials and Methods

### 4.1. Plant Materials and Growth Conditions

The healthy potted Lanzhou lily plants with consistent sizes were chosen for RNA-seq and RT-qPCR experiments. Unopened lily cv. ‘Sorbonne’ flowers of approximately 10 cm in length were selected for transient overexpression and virus-induced gene silencing (VIGS) assays.

### 4.2. De Novo Transcriptome Sequencing and Analysis

After irrigation with distilled water or 2 mg/L TKs at 22 °C for 12 h, Lanzhou lily plants were subjected to 40 °C HS for 0, 6, and 12 h intervals. The total RNA of the middle leaves was extracted using a CTAB-PBIOZOL reagent (Bioflux, Beijing, China). The RNA-seq was performed in the BGISEQ-500 platform (BGI, Shenzhen, China) and the sequencing data are available from NCBI (No. PRJNA1119648). The transcriptome analysis was conducted as described previously [47].

### 4.3. Cloning and Sequence Analysis of LzSCL9

The total RNA extraction from Lanzhou lily leaves and cDNA synthesis were respectively carried out with RNAprep Pure Plant Kit (Tiangen, Beijing, China) and HiScript III 1st Strand cDNA Synthesis Kit (+gDNA wiper) (Vazyme, Nanjing, China). According to our transcriptome sequence, the specific forward primer *LzSCL9*-F and specific reverse primer *LzSCL9*-R were designed for cloning the ORF of *LzSCL9*. The phylogenetic tree was constructed using the neighbor-joining method with 1000 bootstrap replicates in MEGA 7.0 [81]. Multiple alignments of SCL9 amino acid sequences from diverse plant species were conducted using ClustalW 2.0 in BioEdit 7.0 [82]. The primers used for *LzSCL9* cloning are shown in Table 1.

### 4.4. Gene Expression Analysis Using RT-qPCR

Lanzhou lily plants were root-treated with distilled water or 2 mg/L TKs at 22 °C for 12 h, followed by exposure to 40 °C HS for 0, 3, 6, and 12 h. The middle leaves were immediately placed in liquid N_2_ to freeze for RNA extraction. Total RNA isolation and cDNA synthesis were carried out as detailed above. RT-qPCR was conducted to determine *LzSCL9* expression levels with the SYBR Green Supermix (Takara, Dalian, China) on a Roche LightCycler 480 II (Roche, Basel, Switzerland). Lily *18S* rRNA served as reference gene for expression normalization, and primers for RT-qPCR analysis are shown in Table 1.

### 4.5. Transient Overexpression of LzSCL9 in Lily Petals

Based on the methods described by Wu et al. [37], the transient transformation of lily petals was performed with slight modifications. Following resuspension in an infiltration buffer (10 mM MES, 10 mM MgCl_2_, 200 μM acetosyringone, pH 5.6), bacterial cultures expressing SK-II or SK-LzSCL9 were incubated at 22 °C for 3 h in the dark. Lily discs (diameter 1 cm) taken from the inner petals were immersed in bacterial solutions and subsequently vacuum-infiltrated (−0.7 Mpa) for 15 min. The infiltrated discs were rinsed with sterilized water, and then incubated on a 0.4% agar plate at 22 °C for 96 h. The expression level of *LzSCL9* in the infiltrated discs was detected using RT-qPCR analysis. Simultaneously, the discs underwent 40 °C HS for a duration of 12 h. The depigmentation of petal discs and their relative ion leakage were observed and recorded after HS. The primers for constructing the transient overexpression vector are provided in Table 1.

### 4.6. Silencing of LzSCL9 in Lily Petals Using VIGS

The TRV-VIGS procedure was implemented as previously depicted [41] with some minor alterations. A 240 bp fragment of *LzSCL9* was inserted into the TRV2 vector to create TRV2-LzSCL9. Subsequently, TRV1, TRV2, and TRV2-LzSCL9 were individually introduced into *Agrobacterium tumefaciens* GV3101. A mixture of bacterial solutions containing TRV1 and TRV2 or TRV1 and TRV2-LzSCL9 were employed to infiltrate the petal discs as mentioned above. After culturing for 5 days, the infiltrated discs were collected to detect *SCL9* silencing efficiency. Meanwhile, the discs suffered from heat stress (40 °C, 12 h), and were then harvested for the observation of color fading and determination of relative ion leakage. The primers for the construction of the VIGS vector are listed in Table 1.

### 4.7. Statistical Analysis

Student’s *t*-test or Duncan’s multiple range test in SPSS 18.0 was utilized for the statistical analysis of data presented as means ± SD from three replicated experiments. Statistical significance was assigned to a *p*-value lower than 0.05.

## 5. Conclusions

In order to further study the molecular mechanism of TKs in the regulation of the HS tolerance of Lanzhou lilies, we identified a *LzSCL9* gene from the Lanzhou lily that could be induced by heat or heat + TKs treatment in this study. *LzSCL9* expression displayed a significant positive correlation with *LzHsfA2a-1*, and its upregulation was also promoted by TKs during long-term HS. A function analysis demonstrated that lily thermotolerance was increased through *LzSCL9* overexpression and was decreased by *LzSCL9* silencing. In summary, our results indicate that LzSCL9 may be a new positive regulator of TK-elicited thermotolerance in the Lanzhou lily. Our findings also offer a useful candidate gene for molecular breeding for heat tolerance in lilies and other plants.

## Figures and Tables

**Table 1:** The primers used in this article.

Sequences(5′-3′)	
Primers for gene cloning	
*LzSCL9*-F	ATGGTGAACGAGCTAAAAGTTGACGATTT
*LzSCL9*-R	TCATCGACTATTGGGTTTCCACGCAGAGA
Primers for RT-qPCR	
qPCR-*LzSCL9*-F	CTCCATCTGACCAGCCATC
qPCR-*LzSCL9*-R	TAGCGCCAAAGGCCCAATTA
*18S* rRNA-F	AGTTGGTGGAGCGATTTGTCT
*18S* rRNA-R	CCTGTTATTGCCTCAAACTTCC
Primers for transient overexpression	
SK-LzSCL9-XbaI-F	ctccaccgcggtggcggccgctctagaATGGTGAACGAGCTAAAAGTT
SK-LzSCL9-BamHI-R	atatcgaattcctgcagcccgggggatccTCATCGACTATTGGGTTTCCACGC
Primers for VIGS	
TRV2-LzSCL9-XbaI-F	ctgtgagtaaggttaccgaattctctagaTGCGAGGGAACAGA
TRV2-LzSCL9-BamHI-R	gcctcgagacgcgtgagctcggtaccTCATCGACTATTGGGTTTCCACGC

## Data Availability

The original contributions presented in the study are included in the article, further inquiries can be directed to the corresponding author/s.
